# Comparison of accuracy of optic nerve ultrasound for the detection of intracranial hypertension in the setting of acutely fluctuating vs stable intracranial pressure: *post-hoc *analysis of data from a prospective, blinded single center study

**DOI:** 10.1186/CC11336

**Published:** 2012-05-11

**Authors:** Venkatakrishna Rajajee, Jeffrey James Fletcher, Lauryn Renee Rochlen, Teresa Lee Jacobs

**Affiliations:** 1 Department of Neurosurgery, University of Michigan, 3552 Taubman Health Care Center, 1500 E. Medical Center Drive, Ann Arbor, MI 48109-5338, USA; 2 Department of Anesthesia, University of Michigan, University Hospital, 1500 East Medical Center Drive, Room UH1H247, Ann Arbor, MI 48109-5734, USA

## Abstract

**Introduction:**

Optic nerve sheath diameter (ONSD) measurement with bedside ultrasound has been shown in many studies to accurately detect high intracranial pressure (ICP). The accuracy of point-in-time ONSD measurement in the presence of ongoing fluctuation of ICP between high and normal is not known. Recent laboratory investigation suggests that reversal of optic nerve sheath distension may be impaired following bouts of intracranial hypertension. Our objective was to compare the accuracy of ONSD measurement in the setting of fluctuating versus stable ICP.

**Methods:**

This was a retrospective analysis of data from prospective study comparing ONSD to invasive ICP. Patients with invasive ICP monitors in the ICU underwent ONSD measurement with simultaneous blinded recording of ICP from the invasive monitor. Three measurements were made in each eye. Significant acute ICP fluctuation (SAIF) was defined in two different ways; as the presence of ICP both above and below 20 mmHg within a cluster of six measurements (Definition 1) and as a magnitude of fluctuation >10 mmHg within the cluster (Definition 2). The accuracy of point-in-time ONSD measurements for the detection of concurrent ICP >20 mmHg within clusters fulfilling a specific definition of SAIF was compared to the accuracy of ONSD measurements within clusters not fulfilling the particular definition by comparison of independent receiver operating characteristic (ROC) curves.

**Results:**

A total of 613 concurrent ONSD-ICP measurements in 109 clusters were made in 73 patients. Twenty-three (21%) clusters fulfilled SAIF Definition 1 and 17 (16%) SAIF Definition 2. For Definition 1, the difference in the area under the curve (AUC) of ROC curves for groups with and without fluctuation was 0.10 (*P *= 0.0001). There was a fall in the specificity from 98% (95% CI 96 to 99%) to 74% (63 to 83%) and in the positive predictive value from 89% (80 to 95%) to 76% (66 to 84%) with fluctuation. For Definition 2, also, there was a significant difference between the AUC of ROC curves of groups with fluctuation-magnitude >10 mmHg and those with fluctuation-magnitude 5 to 10 mmHg (0.06, *P *= 0.04) as well as <5 mmHg (0.07, *P *= 0.01).

**Conclusions:**

Specificity and PPV of ONSD for ICP >20 mmHg are substantially decreased in patients demonstrating acute fluctuation of ICP between high and normal. This may be because of delayed reversal of nerve sheath distension.

## Introduction

Several clinical studies have now demonstrated that measurement of optic nerve sheath diameter (ONSD) using optic nerve ultrasound (ONUS) may be an accurate and non-invasive bedside tool for the detection of raised intracranial pressure (ICP), typically defined as being >20 or >25 mmHg, when compared to the gold standard of invasive ICP monitoring using intraparenchymal and intraventricular catheters [1-9]. The optic nerve sheath (ONS) is a continuation of the dura, and a significant increase in ICP is transmitted through the subarachnoid space to the ONS as well as the optic nerve head. The eventual distension of the optic nerve head as a result of the transmitted pressure results in papilledema. Papilledema, however, is not a sensitive marker of raised ICP and can take several days to develop [10]. *In vitro *studies suggest that the retrobulbar ONS, on the other hand, may undergo sonographically detectable distension seconds after the development of intracranial hypertension [11,12].

While several studies in the literature have compared the accuracy of ONSD measurement to invasive ICP measurement, there are several unresolved issues that likely need to be addressed before this technique can find more widespread clinical acceptance and use. It may be particularly important to understand the conditions under which ONUS may provide misleading results. The optimal ONSD cut-off for the detection of ICP >20 to 25 mmHg, the confounding effect of retrobulbar hypoechoic artifact, inter-observer variability and the applicability to specific clinical situations are all a matter of debate. Another important and unanswered question is whether ONUS remains accurate in the setting of acutely fluctuating ICP. Since acute fluctuations in ICP between high and normal are common in patients with acute brain injury, particularly in patients with ongoing treatment measures, (such as cerebrospinal fluid removal and the administration of Mannitol), the accuracy of point-in-time ONSD measurement for the detection of true point-in-time intracranial hypertension when ICP ranges between high and low within a short time interval is an issue of significant clinical relevance. The accuracy of ONSD measurement under these circumstances would be heavily dependent on the *in vivo *ability of the ONS to, first, distend immediately following an increase in ICP and then rapidly reverse distension after the ICP drops to normal levels. This issue has not so far been addressed in any published clinical studies. More recently, *in vitro *studies using ONS models have suggested that the ONS may remain distended for a significant period of time following a simulation of raised ICP in the subarachnoid space, even after the reduction of applied pressure to the "normal" range of ICP [13]. While this persistence of ONS distension was primarily observed following elevation of simulated ICP to very high levels (>45 to 55 mmHg) [13], the impact of a possible lag in reversal of ONS distension on the clinical diagnostic accuracy of ONUS is not known.

The recently published ONUS ICP (Optic Nerve Ultrasound for the Detection of Raised Intracranial Pressure) trial was a prospective blinded single-center study conducted at our institution to determine the accuracy of ONSD measurement for the detection of intracranial hypertension in a heterogeneous neuro-ICU population [7]. We conducted *post hoc *analysis of data obtained from this study, to compare the accuracy of ONSD measurement in the setting of stable ICP during the period of evaluation versus in the setting of significant acute ICP fluctuation (SAIF).

## Materials and methods

Approval was obtained from the institutional review board of the University of Michigan (HUM00038460) for the retrospective analysis of data from the database of the ONUS ICP study.

### Inclusion and exclusion criteria

Patients admitted to the neurointensive care unit between November 2008 and May 2011 who had an external ventricular drain (EVD) or intraparenchymal ICP monitor in place and were judged by the treating clinician to be at risk for the development of raised intracranial pressure were enrolled in the ONUS ICP study. Exclusion criteria were age <18 years, known orbital injury and pre-existing optic nerve pathology. Enrollment in ONUS ICP was based on informed consent and investigator availability.

### ONSD and invasive ICP measurement protocol

Blinded ONSD measurements paired with simultaneous measurement of intracranial pressure via invasive monitoring were performed at enrollment and intermittently during the course of the patients' stay in the ICU, based on investigator availability. Most ONSD measurements were performed by a single investigator with three years' experience performing ONUS in a clinical setting (VR), some were performed by a second investigator with two months' experience performing ONUS (JF). The sonographer was blinded to the simultaneous invasive ICP measurement. The bedside monitor was turned away from the sonographer and toward the bedside nurse who recorded the invasive ICP while the ONSD measurement was being performed. The sonographer was, however, not blinded to the patient's diagnosis or clinical history. All EVDs were transduced to the monitor for the duration of each ONSD measurement.

During each evaluation with ONUS, (referred to henceforth as a "measurement cluster"), at least three measurements were attempted from each eye. While each individual ONSD measurement within the measurement cluster was being performed, the minimum and maximum ICP observed on the monitor during the course of the individual measurement was recorded by the bedside nurse and the mean of these two numbers was taken as the corresponding individual invasive ICP measurement. The total time taken to obtain each measurement cluster, that is, six attempted individual measurements, was recorded. Means of the six attempted individual ONSD measurements and of the six attempted individual invasive measurements in each cluster were also recorded; however, individual ONSD measurements were directly correlated to the corresponding individual invasive ICP measurements for assessment of accuracy of ONUS. The use of each of these individual measurements as individual data points for comparison was specifically performed since the primary purpose of this study was to assess the ability of ONSD to change rapidly in reaction to rapidly changing ICP. The presence of widely varying ICP measurements within the cluster of six measurements performed over a significant duration of time means that the most valid comparison to assess the ability of ONSD to keep up with changing ICP was between individual ONSD and ICP measurements within clusters; the mean of the six ONSD measurements would not accurately reflect the ability of a point-in-time ONSD measurement to identify point-in-time intracranial hypertension. Sample clusters are shown in Table 1 (Additional file 1).

All ONUS scans were performed using a point-of-care Sonosite™ M-Turbo (SonoSite Inc., Bothell, WA, USA) ultrasound machine with a 13-6 MHz linear-array probe and a high resolution optimization setting. The probe was placed on the superior and lateral aspect of the orbit against the upper eyelid with the eye closed and angled slightly caudally and medially until the optic nerve was visualized as a linear hypoechoic structure with clearly defined margins posterior to the globe. The probe was always placed gently on the closed eyelid and never in direct contact with the cornea or sclera, to avoid corneal abrasions. Contact with the eye was gentle at all times. Probes and settings specifically approved by the FDA for ocular imaging are recommended. All images were obtained in a transverse/axial plane for uniformity. The ONSD was measured 3 mm behind the retina. In our experience, a major source of error during ONSD measurement is the linear hypoechoic artifact frequently seen posterior to the globe that might be confused with, or partially overlapping, the actual optic nerve sheath. Emphasis was placed on using careful angulation of the transducer during real-time imaging to clearly demarcate the margins of the optic nerve sheath distinct from linear hypoechoic artifact. Images were reviewed off-line while still blinded and the image discarded if the margins of the optic nerve sheath were not sharply defined, there was suspicion of overlap with hypoechoic artifact, or the image was otherwise sub-optimal.

All EVDs were Bactiseal™ (Codman & Shurtleff Inc., Raynham, MA, USA) antimicrobial coated external ventricular drainage catheters; intraparenchymal probes were Codman™ MicroSensor (Codman & Shurtleff Inc.) probes.

### Definitions of SAIF and study groups

We defined significant acute ICP fluctuation (SAIF) in two different ways. In SAIF Definition 1, an ONSD measurement was considered to have been performed in the setting of SAIF when it was part of a measurement cluster in which individual invasive ICP measurements both above and below 20 mmHg were recorded among the six attempted measurements. The purpose of this definition was to capture fluctuation between "high" and "normal" within the period of measurement. To confirm that this definition did in fact result in two groups of measurements performed in settings with significant differences in the *magnitude *of ICP fluctuation (that is, to account for situations in which the ICP may have simply fluctuated in a narrow range around 20 mmHg), we compared the median magnitudes of ICP fluctuation (defined as highest minus the lowest recorded individual invasive ICP measurement within each cluster of measurements) in the two groups and analyzed for a statistically significant difference. Individual ONSD measurements were divided into two groups. ONSD Group 1 consisted of all individual ONSD measurements within those ONSD measurement clusters that fulfilled Definition 1 of SAIF, while ONSD Group 2 consisted of all individual ONSD measurements from those ONSD measurement clusters that did not fulfill the definition. The overall accuracy of each individual ONSD measurement for the detection of corresponding invasive ICP measurement >20 mmHg was compared between Groups 1 and 2 by comparing the results of receiver operating characteristic (ROC) curve analysis performed for each group.

In SAIF Definition 2, an ONSD measurement was considered to have been performed in the setting of SAIF when it was part of a measurement cluster in which the magnitude of ICP fluctuation (defined, as stated earlier, as the highest minus the lowest recorded individual invasive ICP measurement within each cluster of measurements) was >10 mmHg. This definition, therefore, focused on the magnitude of fluctuation over the presence of both high and "normal" within a short period of time. We compared the accuracy of ONSD measurement for the detection of corresponding ICP >20 mmHg between the group of ONSD measurements fulfilling SAIF Definition 2 (Group 1) to the accuracy ONSD measurements performed within clusters in which the magnitude of ICP fluctuation was <5 mmHg (Group 2) and 5 to 10 mmHg (Group 3). This again was done through comparison of the results of ROC curve analysis performed for each group.

Samples of measurement clusters with and without fluctuation per SAIF Definitions 1 and 2 are shown in Table 1 (Additional file 1).

### Effect of ICP spikes and fluctuation within a four-hour period prior to the measurements

In addition to the primary analysis of the effect of SAIF on the accuracy of ONSD measurement, we also studied the effect of spikes in ICP, as well as ICP fluctuation, over a longer (several hours) period prior to study measurements on the accuracy of ONSD measurement. We reviewed the ICP recordings in the patients' medical records for a four-hour period preceding concurrent ONSD and invasive ICP measurement. All ICP spikes to >30 mmHg that persisted for at least five minutes, the lowest ICP in this period and the highest ICP in this period were recorded. The accuracy of ONSD measurements performed within four hours of an ICP elevations to >30 mmHg was compared to the accuracy of ONSD measurements without any preceding spike using independent ROC curves. We separately looked at the range of ICP measurements (highest-lowest recorded ICP) within a four-hour period preceding ONSD measurements. The accuracy of ONSD measurements performed with preceding four-hour ICP ranges of <10 mmHg and >10 mmHg was then compared.

### Statistical analysis

The D'Agostino Pearson test was used to determine if a variable (such as age and time taken to complete concurrent ONSD and ICP measurements) demonstrated normal distribution. Mean with standard deviation and range was calculated for variables with normal distribution and median with interquartile range (IQR) calculated for variables with non-normal distribution. An ROC curve was constructed for each designated group of ONSD measurements, as well the population as a whole, for the ability of ONSD to detect ICP >20 mmHg. The AUC for each ROC curve with 95% CI and standard error (SE) was determined, along with the *P-*value of the AUC being 0.5. We constructed ROC curves for ONSD measurements within clusters fulfilling SAIF Definition 1 (Group 1- ICP both above and under 20 mmHg in the same cluster) and compared the AUC for this ROC curve with the AUC of the ROC curve for all ONSD measurements within clusters not fulfilling SAIF Definition 1 (Group 2) for a statistically significant difference (*P *<0.05). We then constructed an ROC curve for ONSD measurements within clusters fulfilling SAIF Definition 2 (Group 1) and compared the AUC for this ROC curve with the AUC of the ROC curve for all ONSD measurements within clusters with magnitude <5 mmHg (Group 2) and separately for 5 to 10 mmHg (Group 3). A similar analysis was then performed for designated groups of ONSD measurements for the analysis of accuracy in the setting of ICP spikes and fluctuation in the four-hour period preceding the ONSD measurement.

The optimal ONSD cut-off to detect ICP >20 mmHg was determined from each ROC curve. We then calculated the sensitivity, specificity, positive predictive value (PPV) and negative predictive value (NPV) of each cut-off, with 95% confidence intervals (95% CI), for the detection of ICP >20 mmHg by invasive monitoring. *P *<0.05 was also used as the threshold for statistical significance for all the other statistical calculation performed. Statistical analyses were performed using MedCalc for Windows, version 11.6.0.0 (MedCalc software, Mariakerke, Belgium).

## Results

A total of 613 ONSD measurements (in 109 measurement clusters) performed in 73 patients from the ONUS ICP database were included in the analysis. This included 65 patients enrolled in the original ONUS ICP clinical trial [7] and 8 patients who were subsequently prospectively enrolled into the ONUS ICP database following publication of the original study. Overall, 660 measurements were initially performed, of which 47 were discarded following off-line analysis while still blinded, leaving 613 measurements for inclusion in the study. Mean age of the population was 53 + 15 years (range 18 to 90 years). There were 46 women (63%) and 27 men (37%). VR performed 598 (97%) of the measurements while JF performed 18 (3%) of the measurements. The median time taken to complete each cluster of ONSD measurements was 10 minutes (IQR 7 to 14 minutes). Invasive ICP >45 mmHg was seen in one measurement cluster with SAIF and in two measurement clusters without SAIF.

### Diagnostic accuracy of ONSD when acute ICP fluctuation present: Definition 1

Overall, 23/109 (21%) measurement clusters had SAIF per Definition 1. There were 158 ONSD measurements in Group 1 (SAIF Definition 1 present) and 455 measurements in Group 2 (SAIF Definition 1 absent). There was a significant difference (*P *<0.0001) in the median magnitude of ICP fluctuation (highest to lowest invasive ICP within each group of six measurements) between ONSD measurement clusters in Group 1 (12 mmHg, IQR 8 to 19 mmHg) and Group 2 (3 mmHg, IQR 1 to 5 mmHg). Of note, there was a significant difference in the median time taken to complete each measurement cluster between Group 1 (14 minutes, IQR 8 to 19 minutes) and Group 2 (8 minutes, IQR 6 to 11 minutes), *P *= 0.0024.

**Figure 1:**
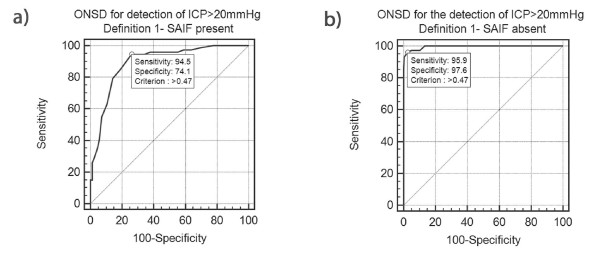
**ROC curves- SAIF Definition 1**. ONSD for the detection of ICP >20 mmHg. Figure 1A represents the ROC for measurements within clusters in which acute fluctuation was present as per SAIF Definition 1, while the ROC curve in figure 1B represents measurements within clusters which did not fulfill SAIF Definition 1.

ROC analysis in Group 1, depicted in Table 2 (Additional file 2) and Figure 1a, revealed AUC = 0.89 (0.83 to 0.94, SE 0.026), *P *(AUC = 0.5) <0.0001. Optimal ONSD for detection of ICP >20 mmHg was >0.48 cm. Sensitivity was 94% (95% CI 87 to 98%), Specificity was 74% (63 to 83%), PPV 76% (66 to 84%) and NPV 94% (85 to 98%). In Group 2, depicted in Table 2 (Additional file 2) and Figure 1b, AUC was 0.99 (0.98 to 1.00, SE 0.0026), *P *(AUC = 0.5) <0.0001, optimal ONSD was >0.48 cm. Sensitivity was 96% (89 to 99%). Specificity was 98% (96 to 99%), PPV 89% (80 to 95%) and NPV 99% (98 to 100%).

The difference between AUCs of the ROC curves constructed for Groups 1 and 2 was 0.10 (*P *= 0.0001).

### Diagnostic accuracy of ONSD when acute ICP fluctuation present: Definition 2

Seventeen of 109 (16%) measurement clusters had SAIF per Definition 2. There were 119 ONSD measurements within clusters with a magnitude of ICP fluctuation >10 mmHg (Group 1, SAIF Definition 2 fulfilled), 142 measurements within clusters with a magnitude of fluctuation 5 to 10 mmHg (Group 2, SAIF Definition 2 not fulfilled) and 352 measurements within clusters with a magnitude of fluctuation <5 mmHg (Group 3, SAIF Definition 2 not fulfilled). There was a significant difference (*P *<0.0001) in the proportion of clusters with recorded individual invasive ICPs both above and below 20 mmHg within the cluster between clusters fulfilling Definition 2 (13/17, 76%) and clusters that did not fulfill Definition 2 (10/92, 11%).

**Figure 2:**
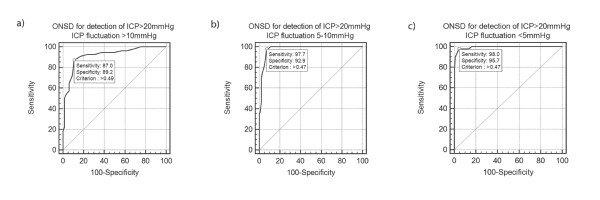
**ROC curves- SAIF Definition 2**. ONSD for the detection of ICP >20 mmHg. Figure 2A represents the ROC for measurements within clusters in which ICP fluctuation>10mmHg was present. Figure 2B represents the ROC for measurements within clusters in which ICP fluctuation was 5-10mmHg. Figure 2C represents the ROC of measurements within clusters in which ICP fluctuation was <5mmHg.

For Definition 2, ROC analysis in Group 1 (Magnitude of fluctuation >10 mmHg), depicted in Table 2 (Additional file 2) and Figure 2a, revealed AUC = 0.92 (0.85 to .96, SE 0.027), *P *(AUC = 0.5) <0.0001. Optimal ONSD for detection of ICP >20 mmHg was >0.50 cm. Sensitivity was 87% (75 to 95%). Specificity was 89% (79 to 96%), PPV was 87% (75 to 95%) and NPV 89% (79 to 96%). When using a >0.48 cm cut-off, Sensitivity was 91% (80 to 97%), Specificity was 83% (72 to 91%), PPV was 82% (70 to 91%) and NPV was 92% (81 to 97%). In Group 2 (Magnitude of fluctuation 5 to 10 mmHg), depicted in Table 2 (Additional file 2) and Figure 2b, AUC was 0.98 (0.94 to 1.00, SE 0.01), *P *(AUC = 0.5) <0.0001 and optimal ONSD >0.48 cm. Sensitivity was 98% (88 to 100%), Specificity was 93% (86 to 97%), PPV 86% (73 to 94%) and NPV 99% (94 to 100%). In Group 3 (Magnitude of fluctuation <5 mmHg), depicted in Table 2 (Additional file 2) and Figure 2c, AUC was 0.99 (0.98 to 1.00, SE 0.004), *P *(AUC = 0.5) <0.0001 and optimal ONSD >0.48 cm. Sensitivity was 98% (89 to 100%), Specificity was 96% (93 to 98%), PPV was 79% (67 to 88%) and NPV 100% (98 to 100%). The differences between AUCs of the ROC curves of Groups 1 and 3 (difference = 0.07, *P *= 0.01) and Groups 1 and 2 (difference = 0.06, *P *= 0.04) attained statistical significance while the difference between the AUCs of the ROC curves of the two groups that did not fulfill Definition 2, Groups 2 and 3, did not (0.01, *P *= 0.35).

### Effect of ICP spikes prior to measurement

Overall, 14 of 109 (13%) measurement clusters were performed within four hours of an ICP spike to >30 mmHg. Analysis of the ROC curve for the measurements within these clusters revealed AUC = 0.97 (0.90 to 1.00, SE = 0.02), *P *(AUC = 0.5) <0.0001. Optimal ONSD for detection of ICP >20 mmHg was >0.49 cm, Sensitivity was 96% (86 to 100%), Specificity was 93% (77 to 99%), PPV was 96% (86 to 100%) and NPV was 93% (77 to 99%). Overall 95 of 109 (87%) measurement clusters did not have an ICP spike to >30 mmHg in the four-hour period preceding measurement. Analysis of the ROC curve for these measurements revealed AUC = 0.97 (0.95 to 0.98, SE = 0.009), *P *(AUC = 0.5) <0.0001. Optimal ONSD for detection of ICP >20 mmHg was >0.48 cm, Sensitivity was 95% (88 to 98%), Specificity was 94% (91 to 96%), PPV was 77% (68 to 84%) and NPV 99% (97 to 100%). There was no difference between the AUCs of the ROC curves of these two curves, suggesting that the presence of ICP spike to >30 mmHg in a four-hour period prior to measurement did not substantially impact accuracy.

### Effect of four-hour ICP fluctuation

The magnitude of ICP fluctuation seen during a four-hour period prior to measurement was >10 mmHg for 314 measurements, performed in 51 of 109 (47%) measurement clusters. The ROC curve for these measurements revealed AUC = 0.95 (0.92 to 0.97, SE = 0.01), *P *(AUC = 0 5) <0.0001. Optimal ONSD for detection of ICP >20 mmHg was >0.47 cm, Sensitivity was 95% (90 to 98%), Specificity was 87% (81 to 92%), PPV was 85% (78 to 90%) and NPV was 96% (91 to 98%). The magnitude of ICP fluctuation seen during a four-hour period prior to measurement was <10 mmHg for 299 measurements, performed in 58 of 109 (53%) measurement clusters. The ROC curve for these measurements revealed AUC = 1.00 (0.98 to 1.00, SE = 0.002), *P *(AUC = 0.5) <0.0001. Optimal ONSD for detection of ICP >20 mmHg was >0.48 cm, Sensitivity was 100% (75 to 100%), Specificity was 99% (98 to 100%), PPV was 87% (58 to 99%) and NPV was 100% (99 to 100%). The difference between the AUCs of the ROC curves for the >10 mmHg fluctuation group and the <10 mmHg fluctuation group was 0.05 and attained statistical significance (*P *= 0.0002). The ability to compare these two groups/ROCs was limited by the fact that very few clusters (only 3 of 58) with a <10 mmHg magnitude of ICP fluctuation contained individual ICP measurements >20 mmHg. There were no recorded ICP measurements >20 mmHg following a four-hour magnitude of ICP fluctuations <5 mmHg.

### Diagnostic accuracy: all measurements

**Figure 3:**
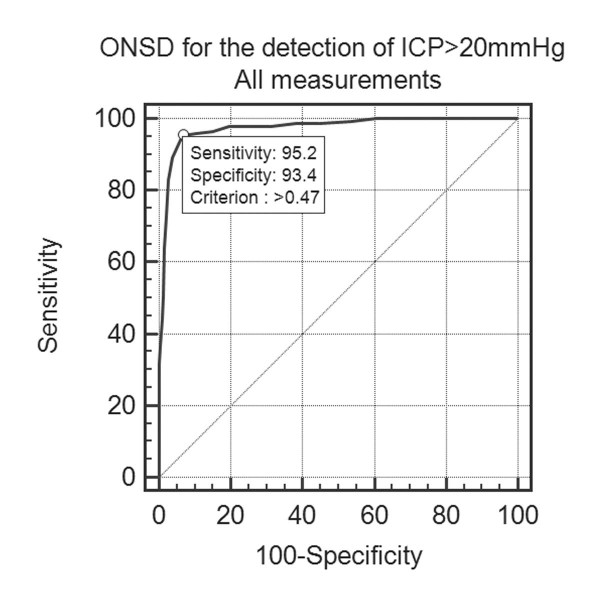
**ROC curve- all measurements**. ONSD for the detection of ICP >20 mmHg- all measurements.

For the population of all ONSD measurements, ROC analysis (Additional file 2: Table 2 and Figure 3) revealed AUC = 0.97 (95% CI 0.96 to 0.99, SE 0.007), *P *(AUC = 0.5) <0.0001. Optimal ONSD for detection of ICP >20 mmHg was >0.48 cm, Sensitivity was 95% (90 to 98%), Specificity was 93% (91 to 96%), PPV was 82% (75 to 87%) and NPV was 98% (97 to 99%).

### Illustrative case description: delayed reversal of ONS distension

**Figures 4:**
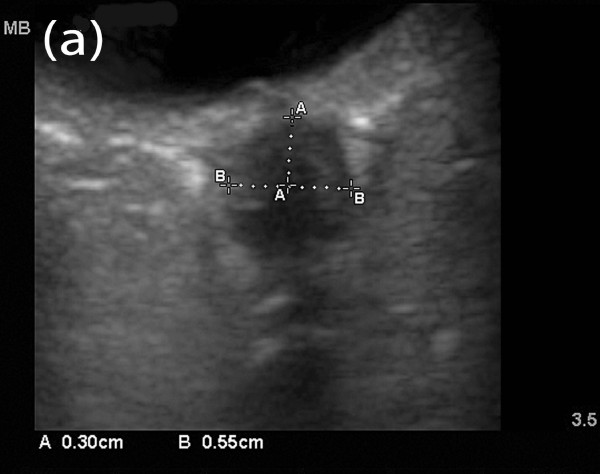
**Delayed reversal of ONS distension- initial scan**. ONUS performed in a 66-year-old patient who became acutely unresponsive two days after removal of an EVD. Scan performed prior to placement of new EVD. The ocular globe is the hypoechoic structure in the upper part of the images and the ONS the linear hypoechoic structure behind the globe. Caliper **A **identifies a point 3 mm behind the retina while Caliper **B **measures the ONSD. ONSD is 0.55 cm, suggestive of raised ICP. Opening pressure was >50 mmHg.

**Figure 5:**
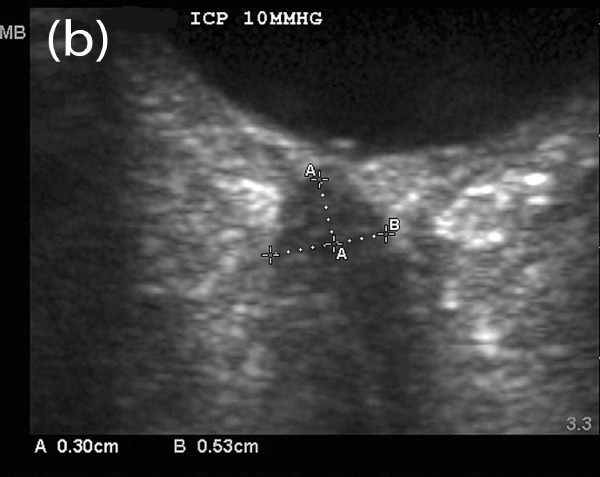
**Delayed reversal of ONS distension- early post-treatment scan**. ONUS performed twenty minutes after first measurement depicted in Figure 4. The ocular globe is the hypoechoic structure in the upper part of the images and the ONS the linear hypoechoic structure behind the globe. Caliper **A **identifies a point 3 mm behind the retina while Caliper **B **measures the ONSD. ONSD is 0.53 cm, suggestive of continued intracranial hypertension. Simultaneously recorded ICP from EVD was only 10 mmHg.

**Figure 6:**
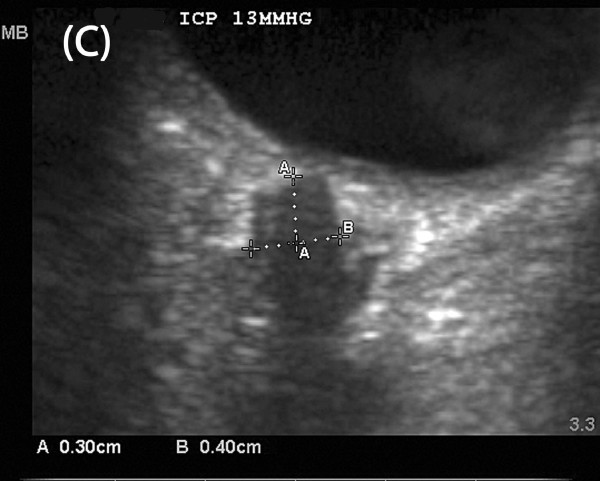
**Delayed reversal of ONS distension- late post-treatment scan**. ONUS performed four hours after first measurement depicted in Figure 4. The ocular globe is the hypoechoic structure in the upper part of the images and the ONS the linear hypoechoic structure behind the globe. Caliper **A **identifies a point 3 mm behind the retina while Caliper **B **measures the ONSD. ONSD is 0.40 cm, suggestive of normal ICP. Simultaneously recorded ICP from EVD was 13 mmHg.

Figures 4 to 6 depict serial ONUS examinations in a patient (not included in the ONUS ICP study) in whom diminished accuracy of ONSD was seen immediately following treatment. A 66-year-old man developed hemorrhage along the EVD following elective resection of a cerebellar mass. He subsequently demonstrated neurological improvement and the EVD was removed after several days. Two days later he became poorly responsive on the general ward, requiring transfer to the ICU and endotracheal intubation for airway protection. CT revealed only mild temporal horn enlargement and ONUS was performed to non-invasively assess for the presence of intracranial hypertension. This revealed an ONSD of 0.55 cm (Figure 4), suggestive of significant intracranial hypertension. An EVD was then placed at the bedside with very high opening pressure (ICP >50 mmHg). Twenty minutes after EVD placement with continuous cerebrospinal fluid drainage, ONUS was repeated, revealing an ONSD of 0.53 cm (Figure 5), again suggestive of raised ICP, despite the measured ICP being only 10 mmHg at this time. When the procedure was repeated four hours later, the ONSD had fallen to 0.40 cm (Figure 6), suggestive of resolution of intracranial hypertension, with the corresponding ICP being 13 mmHg.

## Discussion

The use of ONSD measurement using point-of-care ultrasound for the detection of raised ICP has generated considerable interest, with several studies suggesting a good correlation with the gold standard of invasive ICP monitoring [1-9]. Our study, which includes the largest group of patients (excluding normal controls) in a single study of ONSD to date in the literature, addresses an important potential limitation of ONUS: a possible reduction in diagnostic accuracy in the presence of SAIF. Acute and ongoing ICP fluctuation was seen in about a fifth (16 to 21% depending on the definition used) of all ONUS evaluations in our study, suggesting that this is a fairly common phenomenon and that a decrease in accuracy of the technique in this setting may, in fact, have clinical impact. The fact that 21% of clusters had ICP readings both >20 mmHg and <20 mmHg, and the median 12 mmHg magnitude of ICP fluctuation seen within these clusters, performed over a median duration of 14 minutes, confirms the necessity for the use of individual point-in-time ONSD measurements within clusters as individual data points for comparison with the corresponding individual point-in-time invasive ICP measurements.

In our study, point-in-time ONSD measurement was less accurate for the identification of point-in-time ICP elevation (>20 mmHg) when acute fluctuation was present using two different definitions of SAIF. When the ONSD cut-off for detection of ICP >20 mmHg was kept consistent at >0.48 cm, there was, predominantly, a substantial reduction in the specificity (98% to 74%) and PPV (89% to 76%) when using SAIF Definition 1 (which identified fluctuation between high and "normal" ICP) and a reduction primarily in specificity (96% with a magnitude of fluctuation <5 mmHg to 83% with magnitude of fluctuation >10 mmHg) when using SAIF Definition 2 (which identified a greater absolute magnitude of ICP fluctuation). The implication of this finding is that an ONS that appears to be distended may be less likely to represent a true ICP elevation (decreased PPV) or that fewer true-normal ICPs are correctly identified (decreased specificity) when the ICP is acutely fluctuating. The fact that an apparently distended ONS on sonography is less likely to correlate with high ICP as in the setting of acute fluctuation does raise the possibility that a delay in reversibility of ONS distension may be at least partly responsible. This may be particularly problematic when using ONUS to assess the early response to treatment, as illustrated in the patient described (Figures 4, 5, 6), in which the ONSD continued to suggest significantly elevated ICP even after the ICP had reverted from >50 mmHg to normal with treatment. Of note, while the *in vitro *study of Hansen *et al*. detected a delay in reversibility of ONS distension only after ICP elevation to >45 mmHg [13], we found a diminished accuracy of ONSD measurement in the setting of SAIF despite the presence of very few recorded ICP readings >45 mmHg, in clusters with and without SAIF. In addition, the presence of an ICP spike to >30 mmHg in a four-hour period prior to measurement did not diminish the accuracy of ONSD measurement in our study, while fluctuation between high and normal during the period of measurement (SAIF Definition 1) clearly did. This may suggest that ICP spikes more remote from the time of measurement may be less consequential to accuracy than transient spikes occurring more proximate to, or particularly during, the period of measurement. It is also possible that the decreased accuracy of ONUS in the setting of fluctuation may have primarily been from factors other than a delay in reversibility of ONS distension. In our study, measurement clusters with SAIF took significantly longer to complete than clusters without. This may reflect greater technical difficulty with clearly defining the margins of the ONS on greyscale imaging when ICP and, therefore, the ONSD are actively changing, which may thereby result in diminished accuracy in the presence of SAIF. Our analysis of the effect of ICP fluctuation in a four-hour period prior to measurement did suggest diminished accuracy when the four-hour range of fluctuation was >10 mmHg; however, our ability to perform an accurate statistical analysis of the effect of a greater preceding time period of fluctuation was limited by the fact that ICP elevations to >20 mmHg were quite uncommon when the four-hour range of ICP fluctuation was <10 mmHg.

When using ONUS to assess for the presence of high ICP immediately after treatment, or in other situations where SAIF is likely, performing serial measurements may be useful, especially since ONS distension will almost certainly reverse over time (assuming this is an important cause of the diminished accuracy with fluctuation). It must also be noted that in most clinical situations where there is a high suspicion for the presence of acute intracranial hypertension requiring treatment, an invasive ICP monitor is likely to be placed. Most importantly, however, similar to any other imaging or diagnostic modality used in the ICU, the ONSD must never be used in isolation to make clinical decisions and must always be used in conjunction with all other available clinical data.

There are specific limitations to our study. This study was a retrospective and not a pre-planned analysis. All the data used for analysis were, however, prospectively collected with blinding of invasive ICP. Our study was not primarily directed at studying the phenomenon of delayed reversibility of ONS distension; rather, this is merely one possible explanation for our study's findings related to the actual primary hypothesis- that accuracy of ONUS is diminished when ICP acutely fluctuates. Since our primary stated objective was to analyze the effect of acute fluctuation on accuracy, our definitions of SAIF did not specifically address patients with high ICP being treated, but included all patients with significant fluctuation in either direction. The fact that the specificity and PPV of ONSD were particularly diminished with ICP fluctuation, while the sensitivity and NPV showed only a mild decrease, is suggestive of, but certainly not conclusive evidence for, a problem with reversibility of ONSD distension. Prospective studies with serial ONSD measurement following acute lowering of ICP are required to more specifically address the questions of both the magnitude of inaccuracy following treatment as well as the likely duration of impaired reversal of ONS distension *in vivo*. The available documentation did not permit us to reliably determine the likely proximate etiologies of ICP fluctuation in our study. The presence of a confounding variable responsible for the diminished accuracy of ONSD in patients with SAIF cannot be excluded. The optimal ONSD cutoff for the detection of ICP >20 mmHg overall was significantly lower than the optimal ONSD cutoff proposed by some authors [1,3,5], but similar to that proposed by others [2]. We have previously postulated that the lower optimal ONSD cutoff may be a result of differences in measurement technique and of decreased contamination with retrobulbar hypoechoic artifact [7].

## Conclusions

The specificity and PPV of ONSD measurement for the detection of raised ICP are substantially decreased in the presence of significant acute ICP fluctuation. Delayed reversal of ONS distension is one possible explanation for this finding.

## Key messages

• Optic nerve sheath distension on bedside ultrasound imaging is less predictive of intracranial hypertension when intracranial pressure is acutely fluctuating between high and normal.

• Delayed reversal of optic nerve sheath distension is one possible explanation for this decrease in positive predictive value.

## Abbreviations

AUC: area under curve; BMI: body mass index; CT: computed tomography; EVD: external ventricular drain; ICP: intracranial pressure; NPV: negative predictive value; ONS: optic nerve sheath; ONSD: optic nerve sheath diameter; ONUS: optic nerve ultrasound; PPV: positive predictive value; ROC: receiver operating characteristic; SAIF: significant acute intracranial pressure fluctuation; SE: standard error

## Competing interests

The authors declare that they have no competing interests.

## Authors' contributions

VR conceived of the study, designed the study, acquired data, performed analysis and interpretation of the data, and drafted the manuscript. JFF helped with study design, acquisition of data, analysis of data, critical revision of the manuscript and final approval. LRR helped with acquisition of data, critical revision of the manuscript and final approval. TLJ helped with acquisition of data, critical revision of the manuscript and final approval. All authors have read and approved the manuscript for publication.

## Supplementary Material

Additional file 1 **Table 1 - Clusters of ONSD/ICP measurements: fluctuation vs stable**. Examples of clusters of ONSD measurements with simultaneous invasive ICP measurement. The first two clusters demonstrate SAIF (either by Definition 1 or 2) while the third and fourth do not. Click here for file

Additional file 2 **Table 2 - Accuracy of ONSD for the detection of ICP >20 mmhg - results of ROC analysis**. ROC analysis of different study groups, with and without SAIF. Click here for file
