# Supplementary material for: Comparison of accuracy of optic nerve ultrasound for the detection of intracranial hypertension in the setting of acutely fluctuating vs stable intracranial pressure: post-hoc analysis of data from a prospective, blinded single center study
Source: Crit Care. 2012 May 11;16(3):R79. doi: 10.1186/CC11336 (PMC3580621; doi:10.1186/CC11336)
Supplement: Additional file 1 — Table 1 - Clusters of ONSD/ICP measurements: fluctuation vs stable. Examples of clusters of ONSD measurements with simultaneous invasive ICP measurement. The first two clusters demonstrate SAIF (either by Definition 1 or 2) while the third and fourth do not. [file cc11336-S1.DOC]

**TABLE 1**

**CLUSTERS OF ONSD/ ICP MEASUREMENTS: FLUCTUATION VS STABLE**

**SAMPLE CLUSTER, PATIENT #69: FLUCTUATION PRESENT PER SAIF DEFINTION 1 & 2**

| **TIME** | 14:45 | 14:46 | 14:47 | 14:52 | 14:53 | 14:55 |
| --- | --- | --- | --- | --- | --- | --- |
| **INVASIVE ICP (mmHg)** | 28 | 25 | 21 | 11 | 11 | 13 |
| **INVASIVE ICP (High vs Normal)** | HIGH | HIGH | HIGH | NORMAL | NORMAL | NORMAL |
| **ONSD (cm)** | 0.39 | 0.51 | 0.48 | 0.48 | 0.51 | 0.51 |

This cluster of measurements fulfills SAIF definition 1 since it has ICP measurements both above 20mmHg (at 14:45, 14:46 and 14:48) and below 20mmHg (at 14:52, 14:53 and 14:55). It also fulfills SAIF definition 2 as the magnitude of ICP fluctuation within the cluster is calculated as: 28mmHg (at 14:45) – 11mmHg (at 14:52)= 17mmHg, which is greater than 10mmHg.

SAMPLE CLUSTER, PATIENT #14: FLUCTUATION PRESENT PER SAIF DEFINITION 2 ONLY

| **TIME** | 15:51 | 15:53 | 15:56 | 16:00 | 16:06 | 16:08 |
| --- | --- | --- | --- | --- | --- | --- |
| **INVASIVE ICP (mmHg)** | 29 | 30 | 24 | 35 | 25 | 22 |
| **INVASIVE ICP (High vs Normal)** | HIGH | HIGH | HIGH | HIGH | HIGH | HIGH |
| **ONSD (cm)** | 0.56 | 0.57 | 0.59 | 0.55 | 0.59 | 0.56 |

This cluster does not fulfill SAIF definition 1as no measurement below 20mmHg is present but does fulfill SAIF definition 2 because the magnitude of fluctuation is 35-22=13mmHg, which is >10mmHg.

**SAMPLE CLUSTER, PATIENT #72: FLUCTUATION ABSENT**

| **TIME** | 11:16 | 11:17 | 11:18 | 11:20 | 11:21 | 11:22 |
| --- | --- | --- | --- | --- | --- | --- |
| **INVASIVE ICP (mmHg)** | 7 | 8 | 12 | 9 | 10 | 12 |
| **INVASIVE ICP (High vs Normal)** | NORMAL | NORMAL | NORMAL | NORMAL | NORMAL | NORMAL |
| **ONSD (cm)** | 0.37 | 0.36 | 0.38 | 0.42 | 0.42 | 0.42 |

This cluster fulfills neither criterion for SAIF as no measurement above 20mmHg is present and the magnitude of fluctuation is 12-7=5mmHg, which is <10mmHg.

**SAMPLE CLUSTER, PATIENT #73: FLUCTUATION ABSENT**

| **TIME** | 11:23 | 11:25 | 11:26 | 11:33 | 11:34 | 11:35 |
| --- | --- | --- | --- | --- | --- | --- |
| **INVASIVE ICP (mmHg)** | 22 | 21 | 21 | 25 | 25 | 25 |
| **INVASIVE ICP (High vs Normal)** | HIGH | HIGH | HIGH | HIGH | HIGH | HIGH |
| **ONSD (cm)** | 0.51 | 0.49 | 0.49 | 0.50 | 0.54 | 0.52 |

This cluster fulfills neither criterion for SAIF as no measurement below 20mmHg is present and the magnitude of fluctuation is 12-7=5mmHg, which is <10mmHg.
