# Supplementary material for: Comparison of accuracy of optic nerve ultrasound for the detection of intracranial hypertension in the setting of acutely fluctuating vs stable intracranial pressure: post-hoc analysis of data from a prospective, blinded single center study
Source: Crit Care. 2012 May 11;16(3):R79. doi: 10.1186/CC11336 (PMC3580621; doi:10.1186/CC11336)
Supplement: Additional file 2 — Table 2 - Accuracy of ONSD for the detection of ICP >20 mmhg - results of ROC analysis. ROC analysis of different study groups, with and without SAIF. [file cc11336-S2.DOC]

**TABLE 2**

**ACCURACY OF ONSD FOR THE DETECTION OF ICP>20mmHg- RESULTS OF ROC ANALYSIS**

| **GROUP** | **AUC**  **OF ROC CURVE**  **(95% CI, SE)** | **P (AUC=0.5)** | **ONSD CUT-OFF**  **(cm)** | **SENSITIVITY**  **(95% CI)** | **SPECIFICITY**  **(95% CI)** | **POSITIVE PREDICTIVE VALUE**  **(95% CI)** | **NEGATIVE PREDICTIVE VALUE**  **(95% CI)** |
| --- | --- | --- | --- | --- | --- | --- | --- |
| **SAIF DEFINITION 1- ICP ABOVE AND BELOW 20mmHg IN THE SAME CLUSTER** | | | | | | | |
| **ICP>20 and <20 within cluster PRESENT** | 0.89  (0.83-0.94, SE 0.026) | <0.0001 | >0.48 | 94%  (87-98%) | 74%  (63-83%) | 76%  (66-84%) | 94%  (85-98%) |
| **ICP>20 and <20 within cluster ABSENT** | 0.99  (0.98-1.00, SE 0.0026) | <0.0001 | >0.48 | 96%  (89-99%) | 98%  (96-99%) | 89%  (80-95%) | 99%  (98-100%) |
| **SAIF DEFINITION 2- MAGNITUDE OF ICP FLUCTUATION WITHIN THE CLUSTER** | | | | | | | |
| **Magnitude of Fluctuation**  **>10mmHg** | 0.92  (0.85-0.96, SE 0.027) | <0.0001 | >0.50* | 87%  (75-95%) | 89%  (79-96%) | 87%  (75-95%) | 89%  (79-96%) |
|  |  |  | >0.48* | 91%  (80-97%) | 83%  (72-91%) | 82%  (70-91%) | 92%  (81-97%) |
| **Magnitude of Fluctuation**  **5-10mmHg** | 0.98  (0.94-1.00, SE 0.01) | <0.0001 | >0.48 | 98%  (88-100%) | 93%  (86-97%) | 86%  (73-94%) | 99%  (94-100%) |
| **Magnitude of Fluctuation**  **<5mmHg** | 0.99  (0.98-1.00, SE 0.004) | <0.0001 | >0.48 | 98%  (89-100%) | 96%  (93-98%) | 79%  (67-88%) | 100%  (98-100%) |
| **ALL MEASUREMENTS** | 0.97  (0.96-0.99, SE 0.007) | <0.0001 | >0.48 | 95%  (90-98%) | 93%  (91-96%) | 82%  (75-87%) | 98%  (97-99%) |

*****= The optimal ONSD cut-off for the detection of ICP>20mmHg when the magnitude of fluctuation was >10mmHg was >0.50cm. The accuracy of a >0.48cm cut-off is also displayed on the table for comparison with other groups using a standard cut-off.

**ROC**= Receiver Operating Characteristic curve; **AUC**= Area Under Curve; **SE**= Standard Error; **CI**= Confidence Interval; **ONSD**= Optic Nerve Sheath Interval; **ICP**= Intracranial Pressure
